# GSK‐3β regulates the synaptic expression of NMDA receptors via phosphorylation of phosphatidylinositol 4 kinase type IIα

**DOI:** 10.1111/ejn.14841

**Published:** 2020-06-27

**Authors:** Mascia Amici, Yeseul Lee, Robert J. P. Pope, Clarrisa A. Bradley, Adam Cole, Graham L. Collingridge

**Affiliations:** ^1^ Glutamate Receptor Group School of Physiology, Pharmacology and Neuroscience University of Bristol Bristol UK; ^2^ Lunenfeld‐Tanenbaum Research Institute Mount Sinai Hospital Toronto ON Canada; ^3^ Neurosciences and Mental Health, Peter Gilgan Centre for Research and Learning The Hospital for Sick Children Toronto ON Canada; ^4^ Neurosignalling and Mood Disorders Group Garvan Institute of Medical Research Sydney NSW Australia; ^5^ Tanz Centre for Research in Neurodegenerative Disease, Department of Physiology The University of Toronto Toronto ON Canada

**Keywords:** GSK‐3β, long‐term depression, NMDA receptors, PI4KIIα, rat hippocampus

## Abstract

Deregulation of GSK‐3β is strongly implicated in a variety of serious brain conditions, such as Alzheimer disease, bipolar disorder and schizophrenia. To understand how GSK‐3β becomes dysregulated in these conditions, it is important to understand its physiological functions in the central nervous system. In this context, GSK‐3β plays a role in the induction of NMDA receptor‐dependent long‐term depression (LTD) and several substrates for GSK‐3β have been identified in this form of synaptic plasticity, including KLC‐2, PSD‐95 and tau. Stabilization of NMDA receptors at synapses has also been shown to involve GSK‐3β, but the substrates involved are currently unknown. Recent work has identified phosphatidylinositol 4 kinase type IIα (PI4KIIα) as a neuronal GSK‐3β substrate that can potentially regulate the surface expression of AMPA receptors. In the present study, we investigated the synaptic role of PI4KIIα in organotypic rat hippocampal slices. We found that knockdown of PI4KIIα has no effect on synaptic AMPA receptor‐mediated synaptic transmission but substantially reduces NMDA receptor‐mediated synaptic transmission. Furthermore, the ability of the selective GSK‐3 inhibitor, CT99021, to reduce the amplitude of NMDA receptor‐mediated currents was occluded in shRNA‐PI4KIIα transfected neurons. The effects of knocking down PI4KIIα were fully rescued by a shRNA‐resistant wild‐type construct, but not by a mutant construct that cannot be phosphorylated by GSK‐3β. These data suggest that GSK‐3β phosphorylates PI4KIIα to stabilize NMDA receptors at the synapse.

## Graphical abstract

GSK3β phosphorylates PI4KII⍺ to stabilize synaptic NMDA receptors. .

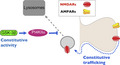

**Abbreviations**
AMPA: α‐amino‐3‐hydroxy‐5‐methyl‐4‐isoxazole propionic acid
EPSC: excitatory post synaptic current
GSK‐3β: glycogen synthase kinase 3 beta
LTD: long‐term depression
NMDA: *N*‐methyl‐*D*‐aspartate
PI4KIIα: phosphatidylinositol 4 kinase type II α

## 1 INTRODUCTION

Glycogen synthase kinase 3 (GSK‐3) is a monomeric, highly conserved, multifunctional Ser/Thr kinase that was discovered for its role in glycogen metabolism (Embi, Rylatt, & Cohen, 1980). The two paralogous proteins GSK‐3α and GSK‐3β are ubiquitously expressed, and many studies have shown that GSK‐3β, in particular, is involved in a plethora of neuronal processes and disorders (Bradley et al., 2012; Kaidanovich‐Beilin & Woodgett, 2011). One of the key features of GSK‐3β is that it is constitutively active (Hur & Zhou, 2010), and most upstream regulators act negatively to reduce GSK‐3β enzymatic activity via an increase of phosphorylation at its Ser9 residue.

Previously we found that the activation of GSK‐3β is involved in NMDA receptor‐dependent long‐term depression (LTD) (Peineau et al., 2007), a form of synaptic plasticity that involves the endocytosis of AMPA receptors and is involved in developmental plasticity and learning and memory (Collingridge, Peineau, Howland, & Wang, 2010). Subsequent work has identified some of the GSK‐3β substrates involved in linking NMDA receptor activation to AMPA receptor endocytosis during LTD; these include kinesis light chain 2 (KLC2) (Du et al., 2010), PSD‐95 (Nelson, Kim, Hsin, Chen, & Sheng,et al., 2013) and tau (Kimura et al., 2014).The regulation of AMPA receptor endocytosis may involve the guanyl nucleotide dissociation inhibitor (GDI):Rab5 complex, whereby activation of GSK‐3β releases Rab5 from GDI such that Rab5 recruits AMPA receptors into early endosomes (Wei et al.,^2010^). In addition to regulating downstream effectors of the LTD induction process, GSK‐3β has also been shown to regulate NMDA receptor function (Chen, Gu, Liu, & Yan, 2007). This process involves regulation of the GDI:Rab5 complex, as well as dynamin and disruption of the binding of NMDA receptors to PSD‐95.

Phosphatidylinositol 4 kinase type II α (PI4KIIα) is highly expressed in the brain where it serves as the major kinase responsible for the formation of phosphatidylinositol‐4‐phosphate (PIP4), a molecule that is involved in many signalling processes. In addition, PI4KIIα phosphorylates a variety of substrates to regulate cellular processes, including membrane trafficking, vesicle trafficking and ion channel function (Minogue, 2018). PI4KIIα is associated, via palmitoylation, with membranes of mainly the trans‐Golgi network and endosomes. In particular, it functions in endosomal sorting processes to direct cargo for lysosomal degradation or recycling to the plasma membrane. Interestingly, PI4KIIα was identified as a neuronal GSK‐3β effector regulating the surface expression of AMPA receptors (Robinson et al., 2014) and endosomal trafficking of AMPA receptors during the reconsolidation of fear memory (Guo et al., 2020). In the present study, therefore, we investigated whether PI4KIIα regulates synaptic AMPA receptors.

We further investigated the possible regulation of synaptic NMDA receptors, and the other major ionotropic subtype expressed at most glutamatergic synapses (Collingridge & Lester, 1989). Hippocampal NMDA receptors are important for both synaptic transmission (Herron, Lester, Coan, & Collingridge, et al., 1986) and synaptic plasticity (Collingridge, Kehl, & McLennan, et al., 1983) and are critically involved in learning and memory (Morris, Hagan, & Rawlins, et al., 1986). Their dysregulation has been implicated in numerous brain disorders, including neurodegenerative conditions, such as Alzheimer's disease (Snyder et al., 2005), psychiatric conditions, such as schizophrenia (Hahn et al., 2006; Mohn, Gainetdinov, Caron, & Koller, 1999) and neurodevelopment conditions, such as autism (Hu, Chen, Myers, Yuan, & Traynelis, 2016). Indeed, NMDA receptors are the likely therapeutic targets of memantine and ketamine in Alzheimer's disease and depression, respectively (Gerhard et al., 2020; Parsons, Stoffler, & Danysz, 2007; Yang, Ju, Zhang, & Sun, 2018). Investigating how NMDA receptors are regulated at synapses provides critical foundations in our understanding of their roles in health and disease.

We used shRNA to knockdown PI4KIIα in hippocampal organotypic slices and studied synaptic transmission and LTD at the Schaffer collateral–commissural pathway. Surprisingly, knockdown of PI4KIIα had no effect on AMPA receptor‐mediated synaptic transmission but substantially reduced NMDA receptor‐mediated synaptic transmission. Pharmacological inhibition of GSK‐3β similarly reduced NMDA receptor‐mediated synaptic transmission, an effect that was precluded by prior knockdown of PI4KIIα. The effects of PI4KIIα knockdown on NMDA receptor‐mediated synaptic transmission were fully rescued by expression of wild‐type PI4KIIα but not by a mutated form of PI4KIIα that is unable to be phosphorylated by GSK‐3β. These results suggest that GSK‐3β phosphorylates PI4KIIα to stabilize NMDA receptors at synapses.

## 2 MATERIALS AND METHODS

### 2.1 Organotypic hippocampal slices

All procedures involving animals were conducted in accordance with the Animal Scientific Procedures Act 1986, UK, and with approval of the University of Bristol. Transverse hippocampal slices from P8 Wistar rats were prepared as described previously (Rocca et al., 2013). Slices were cut in ice‐cold aCSF (in mM: 238 sucrose, 2.5 KCl, 26 NaHCO_3_, 1 NaH_2_PO_4_, 1 CaCl_2_, 9 MgSO_4_, 10 glucose) saturated with 95% O_2_/ 5% CO_2_ then placed on Millicell culture plate inserts (Merck Millipore) and maintained at 35°C, 5% CO_2_ in MEM‐based culture media containing 20% horse serum and (in mM): 30 HEPES, 16.25 glucose, 5 NaHCO_3_, 1 CaCl_2_, 2 MgSO_4_, 0.68 ascorbic acid and 1μg/ml insulin, pH 7.28, 320 mOsm.

### 2.2 Biolistic transfection

Organotypic slices were biolistically transfected at 3–5 days in vitro (DIV) using a Helios GeneGun (Bio‐Rad), and bullets were prepared using PI4KIIα shRNA alone or in combination with either wild‐type or S9/51A shRNA‐resistant PI4KIIα. GFP was used as a transfection marker.

### 2.3 Electrophysiology

Whole‐cell voltage‐clamp recordings were made from CA1 pyramidal cells at 6–11 DIV, blind with respect to the transfected plasmid. Patch pipettes contained intracellular solution (in mM): 8 NaCl, 130 Cs‐methanesulfonate, 10 HEPES, 0.5 EGTA, 4 MgATP, 0.3 Na_3_GTP, 5 QX‐314, pH 7.25, 290 mOsm. Picrotoxin (50 µM) and 2‐chloroadenosine (1 µM) were routinely included in the bath solution, which comprised (in mM): 124 NaCl, 3 KCl, 26 NaHCO_3_, 1.4 NaH_2_PO_4_, 4 CaCl_2_, 4 MgSO_4_, 10 glucose; saturated with 95% O_2_/ 5% CO_2_). In order to isolate NMDA receptor‐mediated EPSCs (EPSC‐N), 3 μM NBQX was added and neurons depolarized to −40 mV. In some cases, D‐AP5 (50 μM) was added at the end of the experiment to confirm synaptic responses were NMDA receptor‐mediated. Two stimulating electrodes (test and control input) were placed in the Schaffer collateral–commissural pathway and stimulated at 0.05 Hz to record AMPA receptor‐mediated EPSCs (EPSC‐A) and at 0.03 Hz for EPSC‐N. Long‐term depression (LTD) was induced using a pairing protocol: 1Hz for 6 min, Vh = −40mV. Data were acquired and analysed with WinLTP (Anderson & Collingridge, 2007). Statistical analysis was performed using paired or unpaired Student's *t* test or one‐way ANOVA as appropriate, and significance was set at *p* < 0.005.

## 3 RESULTS

### 3.1 PI4KIIα knockdown reduces NMDA receptor‐mediated synaptic transmission

To determine whether PI4KIIα regulates the synaptic expression of either AMPA or NMDA receptors, we utilized three previously validated plasmids (Robinson et al., 2014). These were an shRNA probe to reduce the expression of PI4KIIα and two shRNA‐resistant constructs for rescue experiments, a wild‐type (WT) and a mutant PI4KIIα (S9/51A) that cannot be phosphorylated by GSK‐3β. CA1 pyramidal neurons were biolistically transfected with these constructs (plus GFP), and dual whole‐cell patch‐clamp electrophysiological recordings obtained in response to stimulation of the Schaffer collateral–commissural pathway.

**Figure 1:**
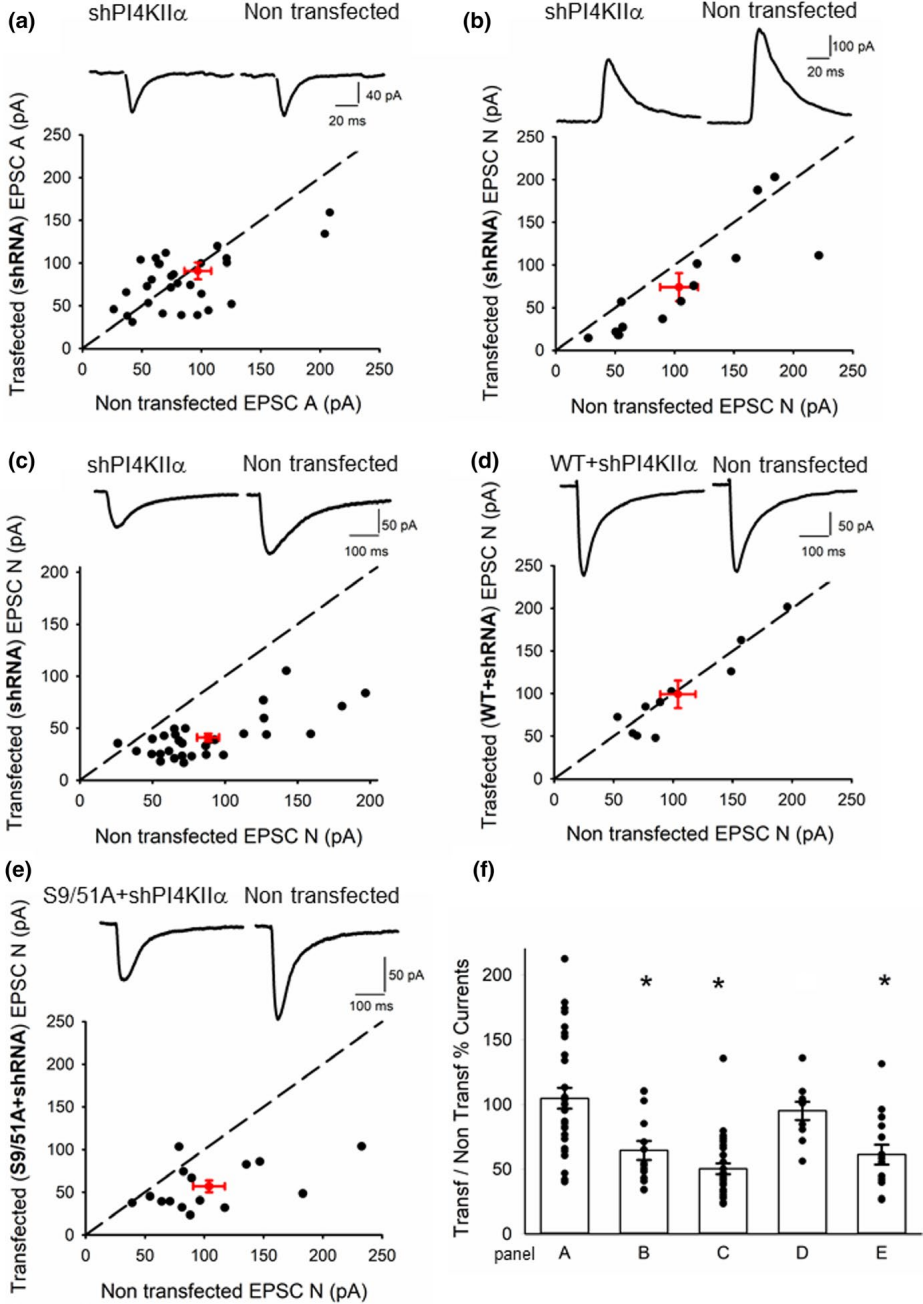
PI4KIIα knockdown reduces NMDA receptor‐mediated synaptic transmission. In graphs (a‐e), EPSCs were recorded in transfected cells and nearby non‐transfected cells, amplitudes were plotted for each pair (black circles), and red circle represents mean ± sem. Insets show representative traces (left: transfected; right: non‐transfected cell). (a) EPSC‐As were recorded in transfected cells (shRNA PI4KIIα) and nearby non‐transfected cells (*n* = 31). (b) EPSC‐Ns were measured 60 ms post‐stimulation at + 40 mV (*n* = 14). (c) In a different set of cells, peak amplitudes in the presence of 3 μM NBQX (at −40 mV) were plotted for each pair (*n* = 30). (d) Pharmacologically isolated EPSC‐Ns were measured in cells transfected with shRNA and WT PI4KIIα and nearby non‐transfected cells (*n* = 10). (e) Pharmacologically isolated EPSC‐Ns were measured in cells transfected with shRNA and S9/51A PI4KIIα and nearby non‐transfected cells (*n* = 15). (f) Summary showing the mean and data points of the ratio of the current amplitude in transfected over paired non‐transfected cell for each condition, * indicates the presence of a statistically significant difference between transfected and non‐transfected cells within a group (labelled after the panels in this figure) following a paired Student's *t* test

Knockdown of PI4KIIα had no effect on AMPA receptor‐mediated excitatory postsynaptic currents (EPSC‐A) as ascertained by comparing the amplitude of the evoked synaptic response in a transfected neuron (91 ± 10 pA) and a nearby non‐transfected neuron (97 ± 11 pA), at a holding potential of −70 mV (105 ± 8% change; *n* = 31 neuronal pairs; Figure 1a,f). In contrast, in the same neurons recorded at + 40 mV, knockdown of PI4KIIα led to a significant reduction in the EPSC (74 ± 16 pA) compared to non‐transfected (104 ± 16 pA) recorded at a latency of 60 ms, at a time when the response is dominated by an NMDA receptor‐mediated component (EPSC‐N, 64 ± 7% change, *n* = 14; Figure 1b,f). To obtain a more accurate estimate of the reduction in EPSC‐N, we pharmacologically isolated the pure NMDA receptor‐mediated synaptic current by blocking the AMPA receptor‐mediated component with NBQX (3 μ M). We made recordings at −40 mV and observed a substantial 50 ± 4% reduction in the amplitude of EPSC‐N in transfected (41 ± 4 pA) compared to non‐transfected (88 ± 8 pA) pairs of neurons (*n* = 30; Figure 1c,f). The effect of the shRNA construct could be ascribed to knockdown of PI4KIIα as there was full rescue in neurons in which an shRNA‐resistant wild‐type PI4KIIα was co‐transfected with the shRNA‐PI4KIIα (99 ± 16 pA) relative to non‐transfected (104 ± 15 pA) neurons (95 ± 7% change, *n* = 10; Figure 1d,f). In contrast, a mutated shRNA‐resistant PI4KIIα that could not be phosphorylated by GSK‐3β (S9/51A) was not able to rescue the reduction in EPSC‐N (57 ± 7 pA) relative to non‐transfected (104 ± 13 pA) neurons (61 ± 8% change, *n* = 15; Figure 1e,f).

### 3.2 PI4KIIα differentially regulates NMDA receptor subtypes

**Figure 2:**
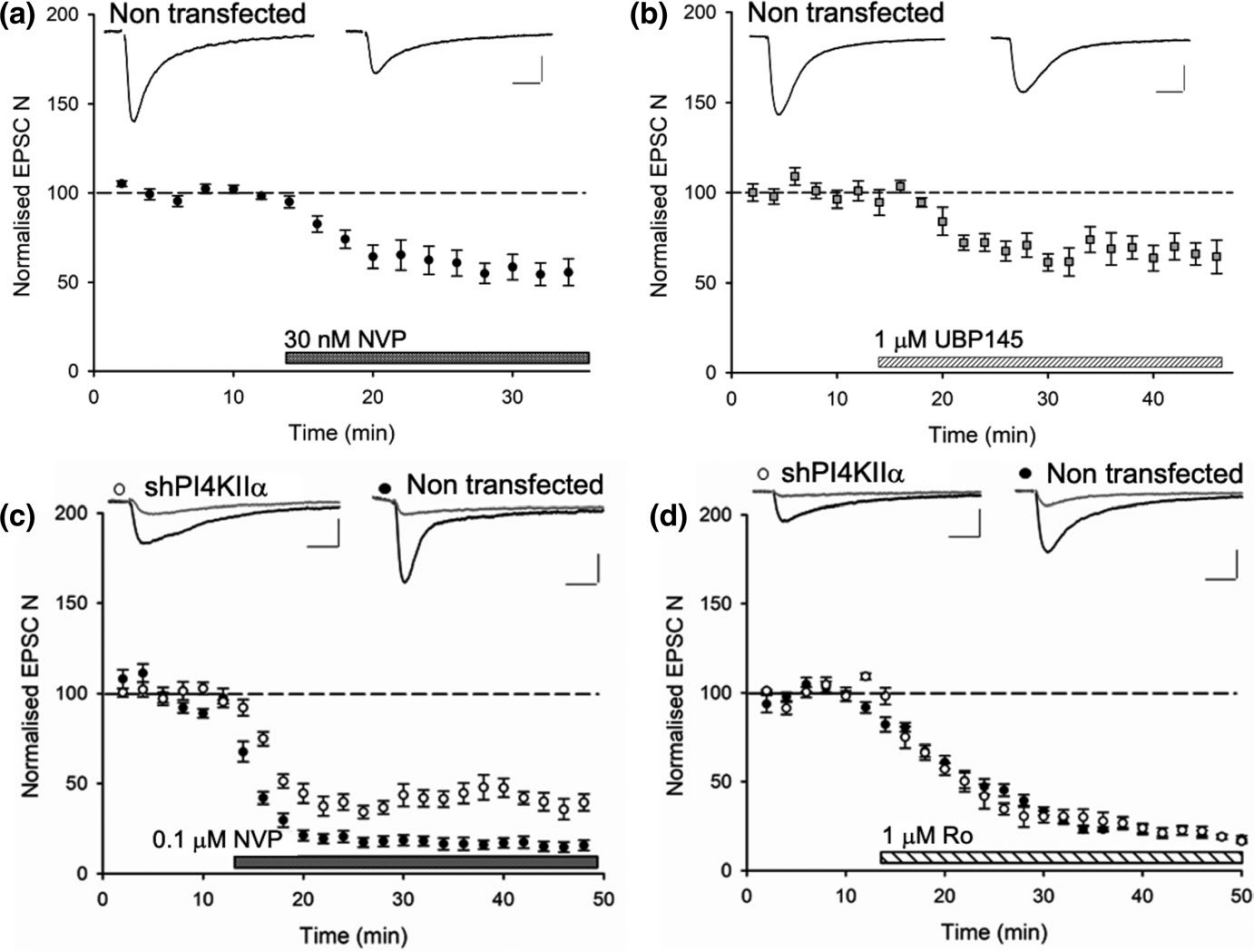
NMDA receptor subtype‐dependent action of PI4KIIα. (a) EPSC‐N responses to NVP‐AAM077 (NVP) at 30 nM concentration in non‐transfected cells (*n* = 10). (b) EPSC‐N responses to 1μM UBP145 in non‐transfected cells (*n* = 4), insets show representative traces before (left) and after application of compounds (right). (c) EPSC‐N responses to NVP at 0.1μM in non‐transfected cells (black circles *n* = 7) and cells transfected with shRNA‐PI4KIIα (white circles *n* = 4; *p* = .002). (d) EPSC‐N responses to Ro25‐6981 (Ro) at 1μM in shRNA‐PI4KIIα‐transfected (white circles *n* = 5) and non‐transfected cells (black circles *n* = 10, *p* = .9). Insets show representative traces before (black) and 30’ after (grey) application of compounds. Calibration bar: 50 pA/ 50 ms

Synaptic NMDA receptors are heteromeric complexes that typically comprise two GluN1 and two GluN2 subunits. The type of GluN2 subunit(s) can determine the trafficking as well as the functional properties of the NMDA receptors. We, therefore, sought to identify the subunit composition of the NMDA receptors that mediate synaptic transmission in organotypic hippocampal slices. We used three antagonists that we have extensively characterized using both recombinant NMDA receptors expressed in HEK cells and native synaptic NMDA receptors in acutely prepared hippocampal slices from adult rat brain (Volianskis et al., 2013) but not hitherto in organotypic slices. The effects of these three NMDAR antagonists on non‐transfected neurons in our organotypic slice preparation are illustrated in Figure 2. NVP‐AAM077 (30 nM and 0.1 μM), which is a GluN2A and GluN2D preferring antagonist, substantially inhibited EPSC‐N to 56 ± 7% at 30 nM (*n* = 10; Figure 2a) and 17 ± 3% at 0.1 μM (*n* = 7; Figure 2c) of baseline. UBP145, which at the concentration used (1 μM) is a selective GluN2D antagonist, also reduced EPSC‐N to 68 ± 9% of baseline (*n* = 4; Figure 2b). Ro25‐6981 (1 μM), which is selective for GluN2B receptors, depressed EPSC‐N to 20 ± 1% (*n* = 10; Figure 2d). These results suggest that GluN2B is a predominant subunit and that GluN2D is also expressed at synapses at this developmental stage.

We compared the ability of two of these antagonists, NVP‐AAM077 (0.1 μM) and Ro25‐6981 (1 μM), to inhibit synaptic transmission in shRNA‐PI4KIIα transfected and non‐transfected neurons. NVP‐AAM077 was less effective in these PI4KIIα knockdown neurons (38 ± 4% transfected, *n* = 4 versus 17 ± 3%; non‐transfected; Figure 2c), whereas Ro25‐6981 was equally effective in both cases (19 ± 2% transfected, *n* = 5 versus 20 ± 1% non‐transfected; Figure 2d). The finding that NVP sensitivity is altered following knockdown of PI4KIIα suggests that it is exerting its effect in an NMDA receptor subunit‐dependent manner.

### 3.3 PI4KIIα is the GSK‐3β downstream effector, which alters EPSC‐N

**Figure 3:**
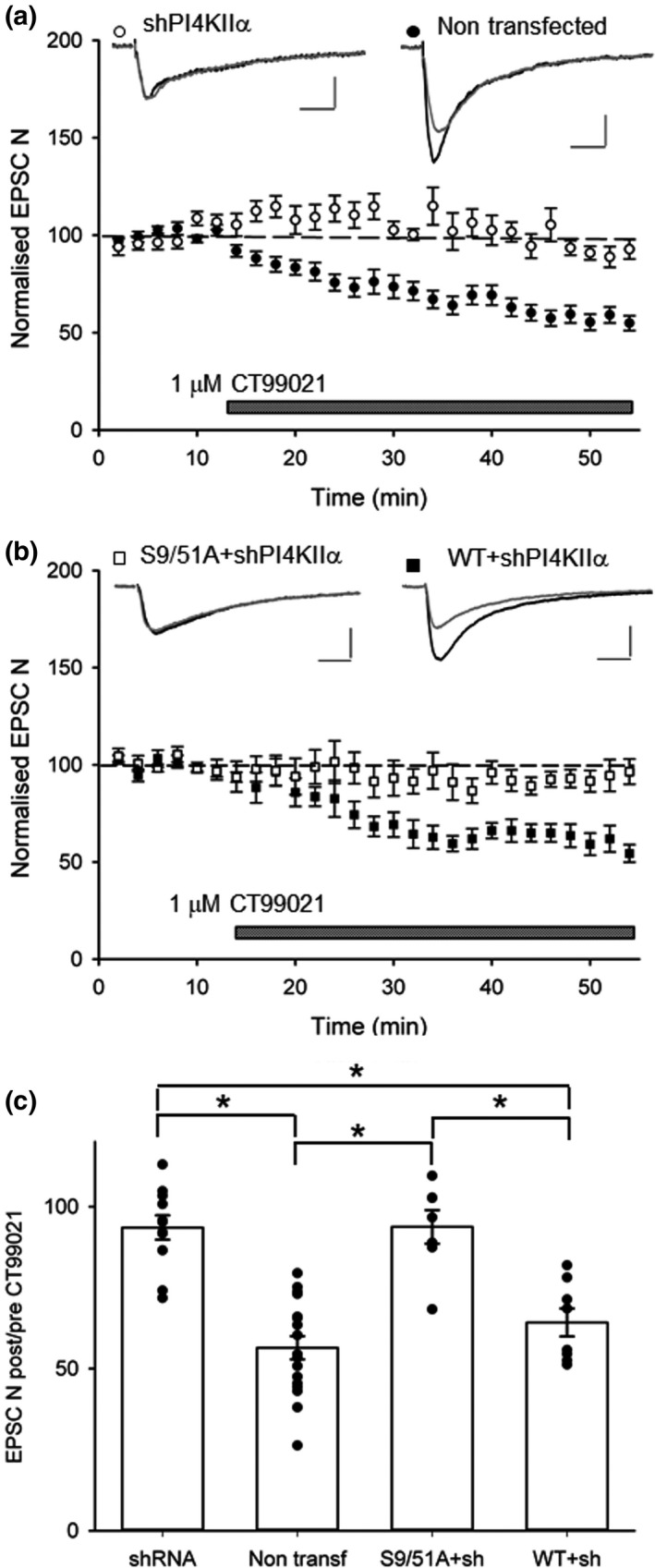
GSK‐3β stabilizes synaptic NMDA receptors via phosphorylation of PI4KIIα. (a) Application of a GSK‐3β inhibitor (CT99021, 1μM) reduced EPSC‐N in non‐transfected cells (black circles *n* = 17), but not in cells transfected with shRNA to knockdown PI4KIIα (white circles *n* = 11). (b) The effect of CT99021 was rescued when wild‐type PI4KIIα shRNA resistant was co‐expressed with the shRNA (black squares *n* = 8), but not when the mutated S9/51A shRNA‐resistant PI4KIIα was co‐expressed with the shRNA (white squares *n* = 7). (c) Summary histograms of the effects of CT99021 (quantified 30 min after start of application) for the various conditions. * indicates the presence of a statistically significant difference between groups. Insets show representative traces before (black) and 30’ after (grey) application of CT99021. Calibration bar: 50 pA/ 50 ms

Previous studies have shown that inhibition of GSK‐3β results in the internalization of NMDA receptors (Chen et al., 2007). Our observation that knockdown of PI4KIIα depresses EPSC‐N suggests that PI4KIIα may be a downstream effector of GSK‐3β in the regulation of synaptic NMDA receptors. To test this directly, we inhibited GSK‐3β using CT99021, its most selective inhibitor (Bain et al., 2007), in control and transfected neurons. CT99021 (1μM) reduced EPSC‐N in control neurons to 57 ± 4% (*n* = 17) of baseline responses but had no effect on neurons transfected by the shRNA‐PI4KIIα construct (94 ± 4%, *n* = 11; Figure 3a,c). The ability of CT99021 to reduce EPSC‐N was rescued in neurons transfected with the wild‐type PI4KIIα construct (64 ± 4%, *n* = 8), but not with the S9/51A mutant construct (94 ± 5%, *n* = 7; Figure 3b,c). Together, these data suggest that GSK‐3β phosphorylates PI4KIIα to stabilize synaptic NMDA receptors.

### 3.4 PI4KIIα is not required for NMDA receptor‐dependent LTD

**Figure 4:**
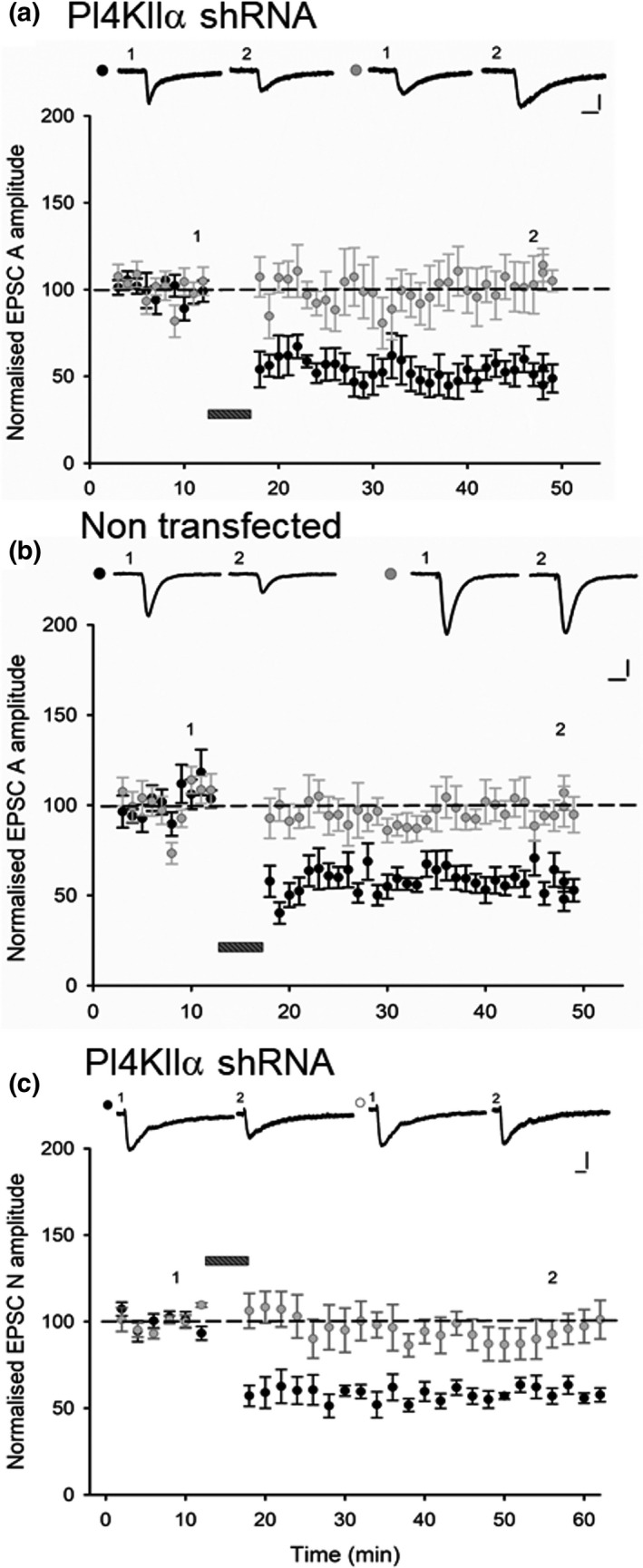
PI4KIIα is not required for NMDA receptor‐dependent LTD. Similar levels of input‐specific LTD were recorded in (a) cells transfected with shRNA‐PI4KIIα (*n* = 8) and (b) non‐ transfected cells (*n* = 7). The bar indicates delivery of a pairing protocol (1 Hz for 6 min, Vh = −40 mV). Representative traces for test (black circle) and control (grey circle) pathway at time points indicated; calibration bar 50 pA/ 20 ms. Percentage of baseline depression of EPSC‐A was quantified 30 min after the pairing protocol. (c) Similar levels of input‐specific LTD of the EPSC‐N were recorded in cells transfected with shRNA‐PI4KIIα (*n* = 4) and control cells (data not shown), depression was quantified 30 min after the pairing protocol. Calibration bar: 30 pA/ 50 ms

Our observations show that knockdown of PI4KIIα expression results in a ~ 50% reduction in the synaptic expression of NMDA receptors. As NMDA receptors are required for the induction of LTD at these synapses, we wondered whether loss of PI4KIIα would impair this process or whether the residual synaptic NMDA receptors could support LTD. To test this possibility, we performed a two pathways experiment, where we used a pairing protocol to induce LTD in the test pathway whilst leaving the control pathway unperturbed (Rocca et al., 2013). We observed similar levels of LTD of EPSC‐A in transfected neurons (54 ± 4% of baseline, *n* = 8; Figure 4a) and non‐transfected neurons (52 ± 7%, *n* = 7; Figure 4b). We also observed a robust LTD of EPSC‐N in transfected neurons (61 ± 4%, *n* = 4; Figure 4c). The induction of LTD of EPSC‐A in transfected neurons was inhibited by D‐AP5 (data not shown) demonstrating the NMDA receptor dependence of this LTD. Therefore, the residual synaptic NMDA receptors are sufficient to enable NMDA receptor‐mediated synaptic plasticity.

## 4 DISCUSSION

In the present study, we have provided evidence that PI4KIIα is required to sustain the full synaptic complement of NMDA receptors. In neurons in which PI4KIIα had been knocked‐down, we found that inhibition of GSK‐3β no longer affected NMDA receptor‐mediated synaptic transmission. Furthermore, we found that this effect was fully rescued by wild‐type PI4KIIα, but not by a mutant form of PI4KIIα that could not be phosphorylated by GSK‐3β. The most straightforward explanation for these results is that GSK‐3β phosphorylates PI4KIIα to maintain a full synaptic complement of NMDA receptors.

### 4.1 PI4KIIα and the regulation of AMPA receptors

We were surprised that knockdown of PI4KIIα had no effect on AMPA receptor‐mediated synaptic transmission given previous work, using the same constructs, had found a modest increase in cell surface AMPARs in cultured hippocampal neurons (Robinson et al., 2014).

**Figure 5:**
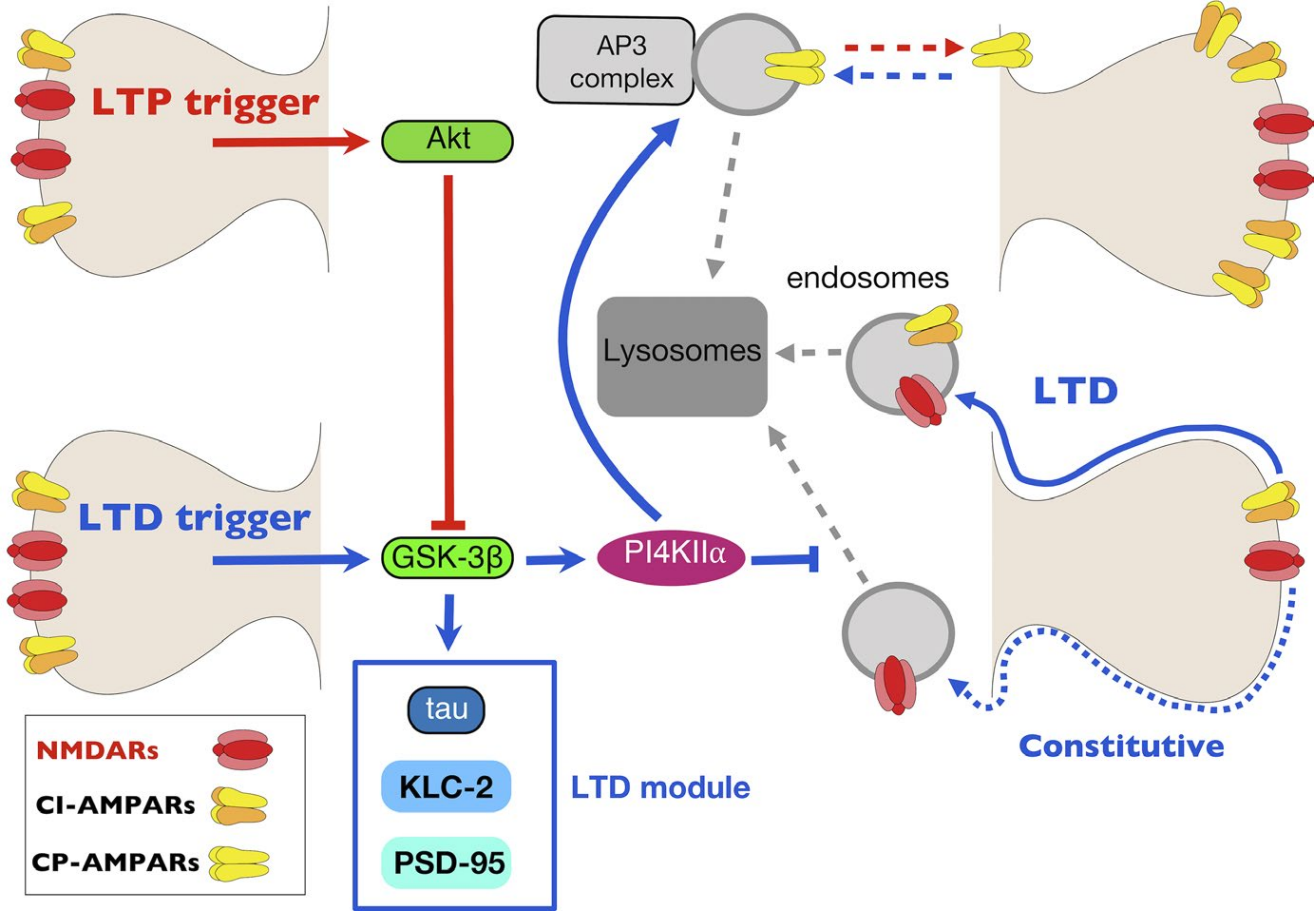
The regulation of glutamate receptor trafficking by PI4KII⍺ during NMDAR‐mediated synaptic plasticity. LTD: (blue arrows). GSK‐3β is activated, and this phosphorylates a number of targets, including tau, KLC2 and PSD95, that result in the endocytosis of GluA2‐containing (i.e. calcium impermeable) AMPARs, via a process that is independent of PI4KII⍺. Additionally, PI4KIIα is phosphorylated by GSK‐3β and this stabilizes a constitutive pool of NMDARs at the synapse. It also drives internalization of GluA2‐lacking (i.e. calcium permeable) AMPARs from extrasynaptic sites, via an AP3‐dependent process, where they are targeted towards lysosomes along with PI4KII⍺ itself. **LTP**: (red arrows). GSK‐3β is inhibited via Akt, and this leads to reduced activity of PI4KII⍺. Accordingly, the internalization of CP‐AMPARs is inhibited leading to their accumulation at extrasynaptic sites

We can envisage at least two possible explanations for this difference. First, it is possible that we had not knocked down PI4KIIα sufficiently to affect AMPA receptor trafficking despite using the same expression constructs as Robinson et al. (2014). The finding that we observe a profound alteration in synaptic NMDARs argues against this possibility. The second possibility is that we studied specifically synaptic AMPA receptors whereas the previous study measured the total complement of cell surface GluA1, which labels both synaptic and extra synaptic AMPA receptors (Richmond et al., 1996). Indeed, GluA1 homomers are preferentially located at extrasynaptic sites and are only driven into these synapses transiently during certain forms of synaptic plasticity (Park et al., 2016). We wondered, therefore, whether an excess of surface GluA1 might result in the appearance of GluA2‐lacking, calcium‐permeable (CP) AMPARs at synapses transfected by the shRNA construct. However, there was no difference in the rectification curves of EPSC‐A in transfected and non‐transfected neurons (unpublished observations). We consider that the most likely explanation for our observations is that PI4KIIα regulates the extrasynaptic pool of GluA1‐containing, GluA2‐lacking CP‐AMPARs but this does not influence the synaptic pool of AMPARs under basal conditions (Figure 5). During the induction of LTP, the activity of GSK‐3β is inhibited (Peineau et al., 2007). Speculatively, this could reduce the PI4KIIα‐mediated trafficking of CP‐AMPARs towards lysosomes and enable the accumulation of newly synthesized CP‐AMPARs at extrasynaptic sites where they would be primed ready for their involvement in LTP (Figure 5).

### 4.2 PI4KIIα and the regulation of NMDA receptors

Previous work had shown that inhibition of GSK‐3β leads to a reduction in the cell surface of NMDA receptors, including NMDA receptor‐mediated synaptic currents (Chen et al., 2007). Our work confirms, using a more specific GSK‐3β inhibitor, CT99021, this effect. The finding that knockdown of PI4KIIα occludes the effect of CT99021 suggests that GSK‐3β is operating via this lipid kinase to mediate this action. Furthermore, the finding that the effect of the knockdown of PI4KIIα on ECPC‐N is fully rescued by a wild‐type PI4KIIα, but not by a mutant PI4KIIα construct, in which the priming sites (Ser9 and Ser51) for GSK‐3β were mutated to alanine, strongly implicates GSK‐3β in the constitutive activation of PI4KIIα, which in turn regulates synaptic NMDA receptors (Figure 5).

In terms of the underlying mechanism, there is evidence that constitutive GSK‐3β activity maintains NMDA receptors on the neuronal plasma membrane by inhibiting their internalization. This clathrin and dynamin‐dependent internalization process involves a Rab5‐directed transport of endocytic vesicles to early endosomes (Chen et al., 2007). It seems likely that PI4KIIα is part of the machinery that links GSK‐3β activity to this intracellular pathway for NMDA receptor removal and degradation.

A major role of PI4KIIα is to bind to AP‐3 on endosomes and catalyse the local formation of PtdIns(4)P, which then recruits additional PI4KIIα and other proteins involved in the formation and targeting of AP‐3 vesicles to lysosomes (Craige, Salazar, & Faundez, 2008). These AP3‐dependent endosomes mediate the movement of multiple membrane proteins to lysosomes for their degradation (Craige et al., 2008; Minogue, 2018; Salazar et al., 2005). However, as we found that knockdown of PI4KIIα led to a reduction in synaptic NMDA receptors, not an increase, we can conclude their constitutive subunits are not cargoes for AP3‐dependent endosomes. In this context, there are a multitude of different cargo adaptor proteins, which permit highly regulated recycling of endocytosed cargoes (Cullen & Steinberg, 2018). Additionally, it is well established that different NMDAR subunits are sorted and processed by various intracellular endocytic pathways (Lavezzari, McCallum, Dewey, & Roche, 2004). We can conclude that whilst the classical role of PI4KIIα in driving endocytic sorting towards lysosomes applies to GluA1 subunits, it does not apply directly to the NMDA receptor subunits that we have investigated here.

A more plausible scenario is that PI4KIIα drives the AP3‐mediated trafficking of proteins, such as dysbindin or the chloride channel CLC‐3 (Farmer, Le, & Nelson, 2013), that limit the functional expression of NMDA receptors (Jeans, Malins, Padamsey, Reinhart, & Emptage, 2011; Tang et al., 2009). For example, dysbindin is thought to be involved in trafficking in the lysosomal pathway and its knockout leads to the accumulation of GluN2A subunits and potentiation of NMDA receptor‐mediated synaptic transmission (Tang et al., 2009). There is also good evidence that PI4KIIα has a role in driving traffic towards the plasma membrane (Minogue, 2018). How precisely PI4KIIα acts to maintain the full complement of NMDA receptors at synaptic sites remains to be determined.

### 4.3 PI4KIIα and NMDA receptor subtypes

Our finding that, in organotypic slices, EPSC‐N is sensitive to low concentrations of Ro25‐6981 and UBP145 implies a contribution of GluN2B and 2D containing NMDA receptors, respectively, to the synaptic response. The sensitivity to NVP‐AAM077 could be explained by an effect on GluN2A and/or GluN2D NMDA receptors at 30 nM with, potentially, some effect on GluN2B at 0.1 μ M. This pharmacological profile is very different from what is observed in adult hippocampal slices, where the predominant NMDA receptor subtype is a GluN2A/GluN2B triheteromer (Volianskis et al., 2013).

The differential effect of NVP in transfected and non‐transfected neurons might be explained by PI4KIIα preferentially stabilizing GluN2A‐containing NMDA receptors, such that its knockdown leads to the reduced sensitivity to NVP‐AAM077 (acting on GluN2A). However, the lack of change in sensitivity to Ro25‐6981 argues against such an action. A more likely possibility is that the synaptic response in these organotypic slices is mainly comprised GluN2B/D triheteromers and GluN2B/B diheteromers. If PI4KIIα preferentially stabilizes GluN2B/2D triheteromers, then its knockdown would lead to a greater proportional content of GluN2B diheteromers reducing sensitivity to NVP‐AAM077 (acting on GluN2D), which is what we observed. The lack of change in sensitivity to Ro25‐6981 is consistent with this model assuming similar sensitivity of GluN2B/2D triheteromers and GluN2B/2B diheteromers, to this class of compound (Yi, Bhattacharya, Thompson, Traynelis, & Hansen, 2019).

The observation that NVP‐AAM077 was less potent on NMDA receptor‐mediated synaptic transmission in slices where PI4KIIα had been knocked down argues for two pools of NMDA receptors at these synapses with differential requirements for PI4KIIα. Had the partial inhibition of synaptic transmission been due to a single population of NMDA receptors and insufficient knockdown of PI4KIIα; then, we would not have expected any alteration in sensitivity to any NMDA receptor antagonist. Rather, our observations favour two pools of synaptic NMDA receptors that are differentially regulated by PI4KIIα. By analogy, synaptic AMPA receptors are also composed of two pools; there is a constitutive recycling pool that is stabilized by an interaction between NSF and the GluA2 subunit and a stable pool that is not regulated in this manner (Nishimune et al., 1998). Further work is required to understand the significance and underlying mechanisms that regulate the PI4KIIα‐dependent and PI4KIIα‐independent synaptic pools of NMDA receptors.

### 4.4 PI4KIIα and the regulation of LTD

An interesting observation was that despite a substantial reduction in the synaptic expression of NMDA receptors, by ~ 50%, NMDA receptor‐mediated LTD was unaffected by PI4KIIα knockdown. First, this shows that PI4KIIα is unlikely to be part of the LTD machinery that links GSK‐3β to AMPA receptor internalization and, hence, distinguishes it from other GSK‐3β substrates that have been directly implicated in this process, such as KLC2 (Du et al., 2010), PSD‐95 (Nelson et al., 2013) and tau (Kimura et al., 2014). Second, it also shows that inhibition of NMDA receptors by GSK‐3β antagonists is unlikely to account for the ability of these antagonists to block the induction of LTD; rather they block the ability of GSK‐3β to regulate the AMPA receptor endocytic machinery directly (Peineau et al., 2007). Third, it suggests the existence of two pools of synaptic NMDA receptors, one regulated by GSK‐3β interacting with PI4KIIα and another that is not, the latter being able to support the induction of NMDA receptor‐dependent LTD (Figure 5). This mechanism would accordingly preserve the bidirectional modifiability of synaptic strength, in the face of a reduction in synaptic NMDA receptors. Future work is required to ascertain whether it is still possible to induce LTP following the knockdown of PI4KIIα.

## 5 CONCLUDING REMARKS

In this study, we have identified a role for the GSK‐3β substrate, PI4KIIα, in the regulation of synaptic NMDA receptors. More specifically, our work has revealed that GSK‐3β phosphorylation of this abundant lipid kinase stabilizes a pool of synaptic NMDA receptors in a subunit dependent manner. Perturbation of this GSK‐3β regulation of PI4KIIα may, therefore, be associated with impaired NMDA receptor function at synapses, which in turn could underlie a plethora of neurological and psychiatric conditions in which NMDA receptors play a critical role.

## CONFLICT OF INTEREST

The authors declare no conflict of interests.

## AUTHOR CONTRIBUTIONS

MA, AC and GLC conceived the project. MA designed and carried out all the experiments and performed the analysis. AC provided the constructs and contributed to the design of the project. YL and RJPP also contributed to the design of experiments. MA, CAB and GLC wrote the paper.

## Data Availability

Data are available upon request from the corresponding author.
